# Supplementary material for: Learning curve and surgical outcome of robotic assisted colorectal surgery with ERAS program
Source: Sci Rep. 2022 Nov 29;12:20566. doi: 10.1038/s41598-022-24665-w (PMC9709162; doi:10.1038/s41598-022-24665-w)
Supplement: Supplementary file 2 — Supplementary Information 2. [file 41598_2022_24665_MOESM2_ESM.zip › RAL Raw data-Ñ[▒K/084/HS_ñw╝╨░O▒Kñσ.pdf]

正式摘要(電子簽章：000000217257966) W92 / W92 ☐英文標題(注意:無英文姓名-請補病人基本檔!)	☐【讀取】憑證	

臺中榮民總醫院
出院病歷摘要

轉入號 : 轉出號 :

[redacted]

**(8)**流水編號	
**(9)**入院日期	110/05/14 (2021) 直腸外科 W92 - 06
**(10)**轉科日期	
**(11)**出院日期	110/05/19 (2021) 住院天數計 5 日

診斷

- 入院 :R-S colon cancer
- 出院 :R-S colon cancer s/p robotic assisted left hemicolectomy on 2021/05/15

主訴

- colon cancer admitted for Robot Assisted Colectomy(L).

病史

- The 38-year-old female had underlying disease of Behcet's disease.
  During health examination, stool OB possitive was noted and after receiving coloscopy, CA, Tis OR T1 OF RS COLON was diagnosed. Therefore, this time, under the impression of colon cancer, she was admitted for Robot Assisted Colectomy(L). She denied any discomfort.

體檢發現

- Anal inspection:
  Recto digital examination:
  Sigmoidoscopy:
  Anoscopy:
  Abdomen:
  - General appearance :
    - easy looking , conscious clear , without cardiopulmonary distress
    - Vital signs自動帶入date 體溫(BT) 脈搏(P) 呼吸(R) 血壓(BP) 身高(BH) 體重(BW) BMI
      20210514 36.1℃ 75次/分 19次/分 121/81mmHg 167.5cm 57kg 20.3
    - Vital signs: B.P. 138/89 mmHg , P.R. 80 /min , R.R. 19 /min , B.T. 36 ℃
  - HEENT :
    - Eyes: no anemic conjunctiva, no icteric sclera, normal EOM
    - ENT: no otorrhea, no rhinorrhea, no erythematous mucosa, no injected tonsil, no deformity
  - Neck: supple, freely movable, trachea midline,no deformity, no engorged jugular vein , no palpable thyroid or lymph node enlargement
  - Chest:
    - Inspection: Grossly normal, symmetric and fully expansion, no accessory muscle used, no operation scar, no spider angiomata, no gynecomastia
    - Palpation: no palpable mass or nodule, no subcutaneous emphysema, no tenderness
    - Ausculation: clear breathing sound
  - Heart:
    - Percussion: normal heart size
    - Ausculation: regular heart beat, normal S1, S2, no gallop, no murmur
  - Abdomen:
    - Inspection:
      - operation scar: .

-
  - Ausculation: normoactive bowel sound, no abdominal bruits
  - palpation: soft, no muscle guarding or rigidity, no local tenderness, no rebounding pain, no palpable mass, no palpable pulsation
  - Percussion: no shifting dulllness, no hepatomegaly, no splenomagaly
-

Peripheral pulsation :	CA	BA	RA	FA	PA	PTA	DPA
Right side :	++	++	++	++	++	++	++/+++
Left side :	++	++	++	++	++	++	++/+++

- Genito-anus: no tenderness or palpable mass lesion or rectal shelf, normal external genitalia, normal anal tone, brown stool

CRS CONDITION

-
  - Lung

手術日期及方法

- 手術日期及方法20210515 1. Robotic (XI) assisted low anterior resection
  2. Intraoperative colonoscopy : (-).
  3. High ligation (+) : At IMA root (Via robotic clips). IMV was ligated via endoclip.
  4. Take down from the splenic flexure : (-)
  5. Robotic EndoGIA (45-4.3) X2 + EEA (33mm) were applied to complete the end to end anastomosis
  6. Proximal cut end: > 10 cm, distal cut end: 1.5 cm
  7. No drainage tube was inserted
  8. ADHESION BARRIER FILM was placed below the umbilicus wound

住院治療經過

- After admission, R-S colon ca for robotic assisted left hemicolectomy was done smoothly on 2021/05/15 under no contraindication of surgery. After operaion, no complication noted and she was discharge under stable condition and arranged OPD followed up.

合併症

一般檢查

- 累積報告 :

DATE	TIME	ALB	TP	BIL.T	BIL.D	ALKP	AST	ALT	LDH	CHOL	TG	HDL-C	LDL	UA	NH3	AMY
1100515	1453	3.6		0.4			11	10								

DATE	TIME	WBC	RBC	HGB	HCT	MCV	PLT	BLST	NEUT	BAND	SEG	LYM	MON	EOS	BAS
1100515	1455	14770		11.3	34.2		289		88.6			7.3	2.7	1.2	0.2

DATE	TIME	NA	K	CL	CA	BUN	CREAT	Mg	P	CRP	ESR	PCT	LIPASE	LACTATE
1100515	1453	141	3.9		8.1	7	0.62							

特殊檢查

病理報告

其它

出院時情況

- 3.改門診治療

出院指示

-

藥名	劑量	劑量單位	用法	頻次	數量	數量單位	現況	天數	單位
(□)Takepron OD tab 30mg	1	TAB	PO	QDAC	7	TAB	TH	7	天
CefaDROxil-500 cap	1	CAP	PO	Q12H	10	CAP	TH	5	天
BethanechoL tab 25mg-JS	1	TAB	PO	TIDAC	21	TAB	TH	7	天
$Biofermin tab	2	TAB	PO	TID	42	TAB	TH	7	天
Ultracet tab	1	TAB	PO	Q6H	28	TAB	TH	7	天

出院計劃

主治醫師_2062F 蔣鋒帆 / 住院醫師_9760F 趙英如

正式摘要**(**電子簽章：**000000217257966)**

W92 / W92

doctorRank:B
